# Assessment of genetic diversity among native Algerian rabbit populations using microsatellite markers

**DOI:** 10.5194/aab-66-207-2023

**Published:** 2023-07-27

**Authors:** Abdelbaki Bouhali, Abdelkader Homrani, Nuno Ferrand, Susana Lopes, Ahmed Mostafa Emam

**Affiliations:** 1 Laboratory of Sciences and Technics for Animal Production (LSTAP), Department of Agronomic Sciences, Faculty of Nature Sciences and Life, Abdelhamid Ibn Badis Mostaganem University, Mostaganem, Algeria; 2 École Normale Supérieur Taleb abderrahmane Laghouat, Laghouat, 4033, Algeria; 3 CIBIO/InBIO, Centro de Investigacao em Biodiversidade e Recursos Geneticos, Campus Agrario de Vairao, Universidade do Porto, 4485-661, Vairão, Portugal; 4 Departamento de Biologia, Faculdade de Ciencias, Universidade do Porto, Rua do Campo Alegre s/n, 4169-007, Porto, Portugal; 5 Animal Production Research Institute, Agricultural Research Centre, Ministry of Agriculture, Nadi El Saiid street, 12618, Dokkii, Giza, Egypt

## Abstract

Having higher adaptability against abiotic stress, which is characterized in rural areas in developing countries, local farm animal genetic resources (FAGRs) are increasingly precarious for random and unsystematic crossing with exotic breeds. In this study, 85 microsatellite loci were utilized to assess genetic diversity among native Algerian rabbits (NARs) sampled from an area of 753 km (from north to south) and 919 km (from east to west). Those distances covered 25 significant geographical points in seven rural areas (El Taref, Mostaganem, Sidi Bel Abbès, M'Sila, Dar Chioukh, Faidh El Botma, and Laghouat). A total of 558 alleles were observed in this study. The highest genetic diversity was registered in the southern direction among NAR populations. The mean number of alleles per locus (MNa) and the inbreeding coefficient ( $F_{\mathrm{IS}}$ ) were highest in Laghouat (4.482 and 0.232), while they were lowest in El Taref (4.000 and 0.149). In the current study, the number of private alleles (Pa) ranged from 9 to 23. In addition, the average of observed heterozygosity (0.427) was lower than the expected value (0.524) due to high levels of inbreeding. The discriminant analysis of principal components (DAPC), the neighbor-joining tree (NJ), and the analysis of STRUCTURE software confirmed the classification of populations according to geographical zones into four main groups (east, west, south, and middle). The results of the current study are useful for breeding improvement and conservation plan research in relation to local animal genetic resources in Algeria.

## 1 Introduction

Algeria is the second leading country in Africa in terms of rabbit meat production, yielding about 8474 t yr ${}^{-1}$ of rabbit meat (FAO, 2021). Moreover, rabbit producers are dependent on exotic rabbit lines commercially for high-production characterization (Berchiche et al., 2012). Gacem and Lebas (2000) and Berchiche et al. (2012) observed that the native Algerian rabbits (NARs) are widely distributed across Algerian rural areas under the backyard and family production systems. In addition, Zerrouki et al. (2005) reported that the NAR adapts differently to abiotic stresses. The phenotype characterizations of NARs are found in several fur colors: black, brown, gray, agouti, white, and distinguished (white with black, brown, and gray), with an average weight of 1.970 kg (Abdelli-Larbi et al., 2014; Mogharbi et al., 2021). The same rabbit phenotype is found in the north African countries of Egypt (Emam et al., 2017; Abdel-Kafy et al., 2018) and Tunisia (Ben Larabi et al., 2014).

Frankham et al. (2002) defined genetic diversity as the total of the alleles and genotypes that influence the morphology, physiology, and behavior of a species. For a very long time, genetic diversity has allowed thousands of domesticated species to adapt to climate, disease, soil characteristics, sources of food, and topography (Hoban et al., 2022). Genetic diversity within and between populations supports ecological functions and provides essential resources and services to humanity (Kettenring et al., 2014; Hollingsworth et al., 2020). Furthermore, diversity studies are very important in achieving the 2030 sustainable-development goals, with number 15 being to halt biodiversity loss.

According to Food and Agriculture Organization (FAO; 2019) statistics, about 15 %–17 % of farm animal genetic resources (FAGRs) are classified as being on the brink of extinction risk. In addition, more than 80 % of livestock breeds are unknown in the Middle Eastern region (FAO, 2015). Genetic diversity contributes to improving the herds of pastoralists and farmers that adapt livestock populations according to environmental conditions and changing demands (Sastry, 2023).

Several types of molecular markers were widely used in rabbit diversity studies, e.g., random amplified polymorphic DNA (RAPD) according to Mohamed and Abdelfattah (2018); the polymerase chain reaction–restriction fragment length polymorphism (PCR-RFLP) by Shevchenko and Kopylov (2015); simple sequence repeat (SSR) microsatellites (Adeolu et al., 2021); the mitochondrial DNA (Emam et al., 2020); and single-nucleotide polymorphisms (SNPs), which were investigated by Ballan et al. (2022). Microsatellite markers are widely used for the naturally codominant, highly polymorphous, and Mendelian inherited (Abdul-Muneer, 2014; Holliday et al., 2018; Karsli et al., 2020; Xia et al., 2021).

On the other hand, the disadvantages of microsatellites are the shadow appearance of stutter bands, the null-allele presence (existing alleles that are not observed using standard assays), and too many alleles at certain loci that would demand a very high sample size for analysis (Abdul-Muneer, 2014). In addition, microsatellite markers sometimes contain mutation lengths that could produce identical-length variants when compromising the population-level studies (Sigsgaard et al., 2020).

In this regard, limited genetic diversity studies were carried out to investigate the NAR genetic situation. For this purpose, the current study is aimed at investigating the genetic diversity of NAR populations at 25 different geographic locations belonging to seven rural regions using 85 microsatellite markers.

## 2 Methods

### 2.1 Rabbit sampling

**Figure 1:**
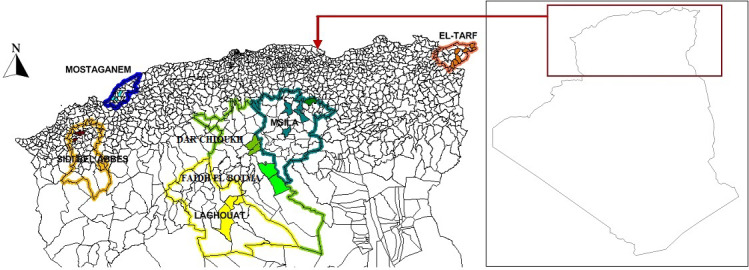
Geographic distribution of sampling strategy. Each population is represented with the same color. The base map of Algeria was downloaded from the web (https://www.geographyknowledge.com/2018/03/Algeria-Blank-Maps.html, last access: 22 December 2022).

**Figure 2:**
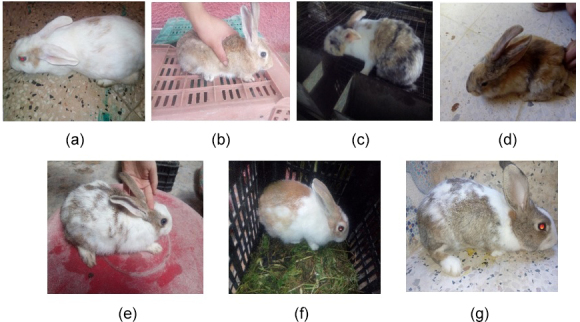
A representative picture of native Algerian rabbits sampled from **(a)** El Taref, **(b)** Mostaganem, **(c)** Sidi Bel Abbès, **(d)** M'Sila, **(e)** Dar Chioukh, **(f)** Faidh El Botma, and **(g)** Laghouat (photos were taken by the authors).

In this study, an Algerian team conducted the survey over about 753 km (from the start point in the north to the end point in the south) and 919 km (from the start point in the east to the end point in the west). A total of 152 tissue samples of NARs were collected from 25 points across seven Algerian rural areas, as shown in Fig. 1, according to FAO conditions (FAO, 2011). The populations were classified according to the following administrative divisions: El Taref (24 samples), Mostaganem (20 samples), Sidi Bel Abbès (22 samples), M'Sila (22 samples), Dar Chioukh (21 samples), Faidh El Botma (22 samples), and Laghouat (21 samples), as shown in Fig. 2. The unrelated animals were sampled from the weaning rabbits and growing rabbits that were prepared for market in the different backyards or from slaughtered rabbits in each geographic location. Tissue samples were stored in 90 % ethanol until DNA extraction was performed.

### 2.2 Laboratory experiment

DNA was extracted from the tissue using DNA EasySpin kits (SP-TD 250, Citomed, Lisbon, Portugal) via the protocol recommended by the manufacturer. The DNA extraction was checked by agarose gel (0.8 %; Nytech 500g, MB 0703). Based on their annealing temperatures, 85 microsatellites were amplified via 15 multiplexes (Table S1 in the Supplement). The multiplex contained 5 µ L of master mix (Qiagen, 20614), 1 µ L of multiplex microsatellite loci (forward-to-reverse primer ratio: 0.1), 1.5 µ L of DNA, and 3 µ L of deuterium-depleted water (dd H ${}_{2}$ O). The PCR products were checked with agarose gel (2 %). The PCR products were migrated on a capillary sequencer (ABI Prism 3310 XL, USA) and scored by GeneMapper 0.4 (Applied Biosystem).

### 2.3 Data analysis

The analysis of molecular variance (AMOVA) and of the estimated number of observed alleles per locus (Na), the mean number of observed alleles (MNa), the total number of private alleles (Pa), and the observed and expected heterozygosity ( $H_{o}$ and $H_{e}$ ) was carried out by GenAlEX 6.41 (Peakall and Smouse, 2012). Cervus 3.0.6 software (Kalinowski et al., 2007) was used to calculate polymorphism information content (PIC), and the Hardy–Weinberg equilibrium (HWE) was used to test significance. The fixation index per population in terms of inbreeding coefficient ( $F_{\mathrm{IS}}$ ) was estimated with 1000 bootstraps using the software GENETIX 4.05 (Belkhir et al., 2004). The discriminant analysis of principal components (DAPC) and the neighbor-joining tree (NJ) were visualized using the R package adegenet V.3.5.0 (R Development Core Team, 2008). The ARES package was used to estimate allelic-richness (Ar) values (Van Loon et al., 2007). The analysis of STRUCTURE software was carried out based on independent runs with 500 000 Markov chain Monte Carlo (MCMC) iterations and a burn-in of 20 000 steps, and this was performed for 1≤K≤10 (Pritchard et al., 2000). The statistic ΔK was computed (Evanno et al., 2005).

## 3 Results

### 3.1 Genetic variability among and within populations

The lowest and highest values in terms of MNa and Pa were detected in El Taref (4 and 9) and Laghouat (4.482 and 23), respectively. $H_{o}$ values ranged from 0.412 (Sidi Bel Abbès) to 0.448 (Faidh El Botma). However, the $H_{e}$ across all the populations varied between Laghouat at 0.543 and El Taref at 0.501. In addition, the $F_{\mathrm{IS}}$ per population was significantly ( P≤0.05 ) higher in the Laghouat population (0.232) than in the El Taref population (0.149). Moreover, the values of Ar varied between 2.913 in Laghouat and 1.833 in El Taref.

A total of 558 alleles were recorded for 85 loci across the populations (Table S2), in which about 19 % of observed alleles were recorded as private alleles ( 106/558 ). The records of Na were varied between 16 and 1 (INRACCDDV0205 and RSPO2, respectively). The highest value of private alleles was recorded in the DRD3 locus (5), while no Pa was recorded in 24 loci (INRACCDDV0108, INRACCDDV0139, INRACCDDV0016, INRACCDDV0140, INRACCDDV0104, INRACCDDV0228, SAT13, GPR64, KLH13, CYTC, HPRT, AMOT, GHRH, IGF1, IGF1R, MSTN, HTR1A, RSPO2, HTRB1B, ESR1, PAX8, ALB, KITLG, and TSHR). Moreover, the values of PIC ranged from 0.083 (ARH) to 0.936 (TCOF1). According to PIC values (Table S2), the majority of studied loci ( 49/85 loci) showed a highly informative expression ( PIC>0.5 ), while 25 loci showed moderately informative expression ( 0.25<PIC<0.5 ), and 5 loci showed a lowly informative expression ( 0.25>PIC ). The percent of HWE significance was 89.9 % with different levels ( P<0.05,P<0.01 , and P<0.001 ) when 10.1 % of the loci did not express significance.

### 3.2 The genetic differentiation among NARs

**Figure 3:**
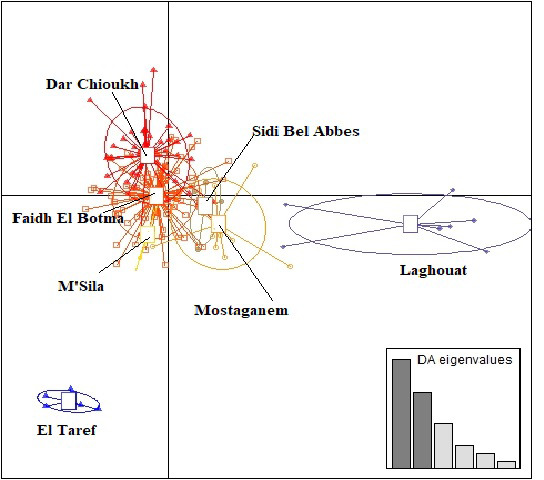
Discriminant analysis of principal components (DAPC) of seven native Algerian rabbit populations, where the horizontal axis represents the first linear discriminant and the vertical axis represents the second linear discriminant.

**Figure 4:**
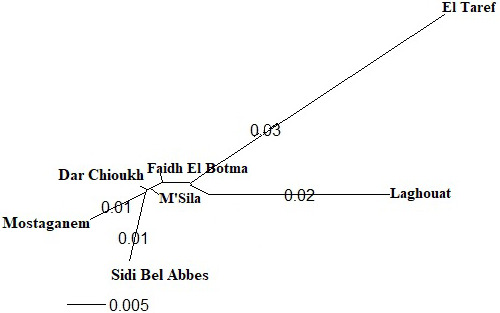
The phylogenetic tree constructed from Nei's standard genetic distances of seven native Algerian rabbit populations.

Figure 3 presents the result of DAPC analysis among NAR populations. There was convergence among the middle zones (Dar Chioukh, Faidh El Botma, and M'Sila) with the southern (Laghouat) and the western zones (Mostaganem and Sidi Bel Abbès), while the eastern zone (El Taref) was expressed as being far. The same concept is found in the NJ tree (Fig. 4).

**Figure 5:**
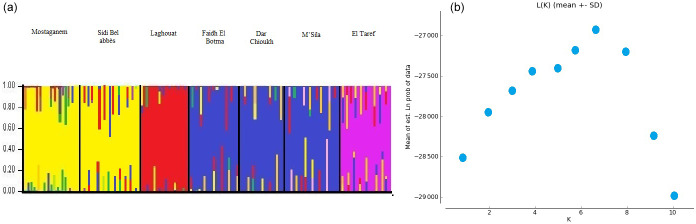
**(a)** Genetic structure of native Algerian rabbit populations inferred by Bayesian analysis at K=7 using STRUCTURE software; **(b)** ΔK calculated from relation of K=1 to K=10 between populations. K refers to the number of assumed clusters.

The STRUCTURE analysis and values of ΔK of NARs are shown in Fig. 5a and b. The highest values of ΔK were obtained when K=7 (Fig. 5b). Two populations in the east and south were expressed in separate clusters (El Taref and Laghouat, respectively), whereas the middle populations (M'Sila, Dar Chioukh, and Faidh El Botma) were clustered together. Also, the Mostaganem and Sidi Bel Abbès populations in the west were clustered together.

### 3.3 Analysis of molecular variance for NAR

The results of AMOVA are summarized in Table 2. It is shown that the measured genetic differentiations of the total genetic variance among populations and individuals were 3 % and 20 %. However, the variation of within-population genetic diversity was 76 %.

## 4 Discussion

As shown in Table 1, the mean values for MNa and Pa in NAR populations were 4.277 and 15.14, respectively. This result is consistent with the findings of Bolet et al. (2000) and Alves et al. (2015) for European domestic rabbits; they stated mean values for MNa of 3.600 and 3.156, respectively. In addition, this study result is nearly similar to the values recorded in north African regions such as Egypt and Tunisia. In this regard, in Egypt, Emam et al. (2017) found a high MNa that ranged from 4.316 to 6.000, while recorded values ranged between 5 and 15 for Pa. On the other hand, in Tunisia, Ben Larabi et al. (2014) reported low values of MNa that ranged from 3.000 to 4.370. Moreover, in this study, the $H_{e}>H_{o}$ and $F_{\mathrm{IS}}$ values were recorded as positive. This result is in agreement with Ben Larabi et al. (2014), Emam et al. (2017), and Fouzia et al. (2017). This is considered to be a strong indicator of strong inbreeding, as mentioned by Schmidt et al. (2021). Furthermore, the value of Ar is a good reflection of mutation (Ali et al., 2018). In the current study, the highest diversity values were observed towards the southward direction (Laghouat, Faidh El Botma, and Dar Chioukh populations). This is maybe due to the high temperatures, which cause an increase in mutations (Woldvogel and Pfenninger, 2021), and the mutation is the intrinsic reason for increasing the diversity (Teixeira and Huber, 2021). This result is proven by the findings of Emam et al. (2017), who found high diversity in local Egyptian rabbits in the south (Emam et al., 2017). In the same context, Zeroual et al. (2020) reported that the temperature is significantly hotter in the south of Algeria than in the north.

**Table 1:** Genetic diversity parameters in native Algerian rabbit populations.

Populations	MNa ± SE	Pa	$H_{o}$ ± SE	$H_{e}$ ± SE	Ar ± SE	$F_{\mathrm{IS}}$
El Taref	4.000 ± 0.190	9	0.425 ± 0.022	0.501 ± 0.023	1.833 ± 0.142	0.149 ${}^{\text{f}}$
Mostaganem	4.035 ± 0.158	11	0.433 ± 0.022	0.521 ± 0.021	2.003 ± 0.157	0.166 ${}^{\text{e}}$
Sidi Bel Abbès	4.329 ± 0.206	17	0.412 ± 0.023	0.522 ± 0.022	2.386 ± 0.214	0.182 ${}^{\text{d}}$
M'Sila	4.259 ± 0.200	14	0.438 ± 0.021	0.534 ± 0.022	2.111 ± 0.173	0.170 ${}^{\text{e}}$
Dar Chioukh	4.400 ± 0.185	15	0.389 ± 0.022	0.513 ± 0.023	2.542 ± 0.199	0.197 ${}^{\text{c}}$
Faidh El Botma	4.435 ± 0.181	17	0.448 ± 0.024	0.536 ± 0.023	2.661 ± 0.222	0.213 ${}^{\text{b}}$
Laghouat	4.482 ± 0.193	23	0.441 ± 0.024	0.543 ± 0.023	2.913 ± 0.210	0.232 ${}^{\text{a}}$
Mean value	4.277 ± 0.071	15.14	0.427 ± 0.008	0.524 ± 0.008	2.349 ± 0.188	0.187

Mean number of observed alleles (MNa), standard error (SE), number of private alleles (Pa), mean observed and expected heterozygosity ( $H_{o}$ and $H_{e}$ ), allelic richness (Ar), and inbreeding coefficient ( $F_{\mathrm{IS}}$ ). Values followed by different superscripts, (a, b, c, d, e, and f) within the last column are significantly different ( P≥0.05 ).

Moreover, results in Table S2 showed that 57 % of loci were highly formative in terms of PIC values. The high percentage of formative PIC was also observed in several studies (Alves et al., 2015; El-Aksher et al., 2016; Lai et al., 2018). On the other hand, 89 % of the loci were significant in terms of HWE at three significance levels ( P<0.05,P<0.01 , and P<0.001 ), which is generally characteristic for an inbreeding situation in NARs. In contrast, the values recorded in the commercial rabbits in Nigeria were not significant (Adeolu et al., 2021).

The DAPC (Fig. 3) allowed us to classify NAR populations into four major groups according to geographical groups (eastern, western, southern, and middle populations). The geographical integration was found between the middle populations (M'Sila, Faidh El Botma, and Dar Chioukh). It is due to the geographical proximity of those areas (less than 150 km). On the other hand, the overlap between the middle and southern populations could be explained by the distance between the Laghouat population (in the south) and the Faidh El Botma population (the last city in the middle zone), which is less than 110 km. In addition, the distance between the western points (Mostaganem and Sidi Bel Abbès) is less than 130 km. In contrast, the eastern population was expressed as being far separated from the western (919 km), middle (635 km), and southern (753 km) populations. The geographical isolation was found for wild rabbits (Fuller et al., 1997; Carneiro et al., 2013; Alda and Doadrio, 2014). In addition, the rabbit population classification according to the geographical zones was reported in wild rabbits (Carneiro et al., 2013; Alda and Doadrio, 2014; Iannella et al., 2019; Alves et al., 2022) and local rabbit breeds (Ben Larabi et al., 2012, 2014; Emam et al., 2016, 2017; Jochová et al., 2017). The same results were recorded in the NJ tree (Fig. 4).

According to the analysis of STRUCTURE (Fig. 5a and b), more populations were clustered together in the middle and west (Fig. 5a). Emam et al. (2016) reported that more populations clustered together in the Egyptian local breeds, while other populations were separated in each cluster (in the east and south), which confirmed the results in Figs. 3 and 4. The most likely values of ΔK were obtained for K=7 (Fig. 5b). The highest value of ΔK was equal to population number, in agreement with Ben Larabi et al. (2014), Emam et al. (2016, 2017), and Dudu et al. (2020).

The results of AMOVA (Table 2) showed a high value of genetic variation among individuals (20 %), which is a strong indicator for permitting flexibility and the survival of a population in the face of changing environmental circumstances (Pavlova et al., 2017; Ma et al., 2020). On the other hand, the among-population variation (3 %) is a strong indicator of the closing inbreeding system and could be the direct result of the infrequent gene flow that increased the chance of recombination (Bortoluzzi et al., 2018; Núñez-Torres and Almeida-Secaira, 2022). In contrast, El-Aksher et al. (2016) in Egypt and Adeolu et al. (2021) in Nigeria found random breeding systems with lower values among populations (1 %) and individuals (4 %) in commercial rabbit lines.

**Table 2:** Summary table for analysis of molecular variance (AMOVA).

Source	df's	SS	MS	Est. var.	% var.
Among populations	6	359.959	59.993	0.741	3 %
Among individuals	145	4033.426	27.817	4.848	20 %
Within individuals	152	2754.500	18.122	18.122	76 %
Total	303	7147.885		23.710	100 %

Degrees of freedom (df's), sum of squares (SS), mean square (MS), estimated variance (Est. var.), and percentage of variation (% var.).

## 5 Conclusions

The current study is the first study dependent on using a large number of microsatellite loci to understand the genetic diversity of native Algerian rabbit populations. According to the discovered results, high diversity was recorded in the south. Also, a high degree of geographical distribution (east, west, middle, and south) was noticed in the results. Generally, the current study records a high internal-breeding factor, although the samples were collected randomly. This is a strong indicator that native Algerian rabbits are in an endangered situation. This study could be used as a guide for rabbit genetic improvement and conservation strategies in Algeria. The results of this study could open up areas for cooperation among north African countries by studying the genetic diversity of native rabbits. In the same context, cooperation could be included to design maintenance programs (genetic improvement and conservation) for native rabbit breeds, which could facilitate sustainable rural development plans in this important region. It will meet goal 15 (to halt biodiversity loss) of the United Nations' sustainable development agenda for 2030.

## Supplement

10.5194/aab-66-207-2023-supplement The supplement related to this article is available online at: https://doi.org/10.5194/aab-66-207-2023-supplement.

## Data Availability

The datasets are available upon request from the corresponding author.

## References

[bib1.bib1] Abdelli-Larbi O, Mazouzi-Hadid F, Berchiche M, Bolet G, Garreau H, Lebas F (2014). Pre-weaning growth performance of kits of a local Algerian rabbit population: influence of dam coat color, parity and kindling season. World Rabbit Sci.

[bib1.bib2] Abdel-Kafy EM, Ahmed SS, El-keredy A, Ali NI, Ramadan S, Farid A (2018). Genetic and phenotypic characterization of the native rabbits in Middle Egypt. Veterinary World.

[bib1.bib3] Abdul-Muneer PM (2014). Application of microsatellite markers in conservation genetics and fisheries management: recent advances in population structure analysis and conservation strategies. Genet Res Int.

[bib1.bib4] Adeolu AI, Wheto M, Oleforuh-Okolehc VU, Nwose RN, Adenaike AS, Yakubu A, Abiola EM, Mohammed BG (2021). Genetic Diversity of Rabbit *(Oryctolagus cuniculus)* Population in South Eastern Nigeria Using Microsatellite Markers. Tropical Animal Science Journal.

[bib1.bib5] Alda F, Doadrio I (2014). Spatial genetic structure across a hybrid zone between European rabbit subspecies. PeerJ.

[bib1.bib6] Ali Q, Rashid I, Shabbir MZ, Shahzad K, Ashraf K, Sargison ND, Chaudhry U (2018). Population genetics of benzimidazole-resistant Haemonchus contortus and Haemonchus placei from buffalo and cattle: implications for the emergence and spread of resistance mutations. Parasitol Res.

[bib1.bib7] Alves JM, Carneiro M, Afonso S, Lopes S, Garreau H, Boucher S, Allian D, Queney G, Esteves PJ, Bolet J, Ferrnand N (2015). Levels and patterns of genetic diversity and population structure in domestic rabbits. PLoS One.

[bib1.bib8] Alves JM, Carneiroa M, Day JP, Welch JJ, Duckworthe JA, Cox TE, Letnic M, Strive T, Ferranda N, Jiggins FM (2022). A single introduction of wild rabbits triggered the biological invasion of Australia. P Natl Acad Sci USA.

[bib1.bib9] Ballan M, Bovo S, Schiavo G, Schiavitto M, Negrini R, Fontanesi L (2022). Genomic diversity and signatures of selection in meat and fancy rabbit breeds based on high-density marker data. Genet Sel Evol.

[bib1.bib10] Belkhir K, Borsa P, Chikhi L, Raufaste N, Bonhomme F (2004). GENETIX 4.05, logiciel sous Windows TM pour la génétique des populations.

[bib1.bib11] Ben Larabi M, San-Cristobal M, Chantry-Darmon C, Bolet G (2012). Genetic diversity of rabbit populations in Tunisia using microsatellites markers.

[bib1.bib12] Ben Larabi M, San-Cristobal M, Chantry-Darmon C, Bolet G (2014). Population structure in Tunisian indigenous rabbit as curtained using molecular information. World Rabbit Sci.

[bib1.bib13] Berchiche M, Cherfaoui D, Lounaouci G, Kadi SA (2012). Utilisation de lapins de population locale en élevage rationnel: Aperçu des performances de reproduction et de croissance en Algérie.

[bib1.bib14] Bolet G, Brun JM, Monnerot M, Abeni F, Arnal C, Arnold J, Bell D, Bergoglio G, Besenfelder U, Bosze S, Boucher S, Chanteloup N, Ducourouble MC, Durand-Tardif M, Esteves PJ, Ferrand N, Gautier A, Haas C, Hewitt G, Jehl N, Joly T, Koehl PF, Laube T, Lechevestrier S, Lopez M, Masoero G, Menigoz JJ, Piccinin R, Queney G, Saleil G, Surridge A, Van Der Loo W, Vicente JS, Viudes De Castro MP, Virag G, Zimmermann JM (2000). Evaluation and conservation of European rabbit (*Oryctolagus cuniculus*) genetic resources, First results and inferences.

[bib1.bib15] Bortoluzzi C, Crooijmans RPMA, Bosse M, Hiemstra SJ, Groenen MAM, Megens HJ (2018). The effects of recent changes in breeding preferences on maintaining traditional Dutch chicken genomic diversity. Heredity.

[bib1.bib16] Carneiro M, Baird SJE, Afonso S, Ramirez E, Tarroso P, Teotonio H, Villafuerte R, Nachman MW, Ferrand N (2013). Steep clines within a highly permeable genome across a hybrid zone between two subspecies of the European rabbit. Mol Ecol.

[bib1.bib17] Dudu A, Popa G-O, Ghiță E, Pelmuș R, Lazăr C, Costache M, Georgescu SE (2020). Assessment of genetic diversity in main local sheep breeds from Romania using microsatellite markers. Arch Anim Breed.

[bib1.bib18] El-Aksher SH, Sherif HS, Khalil MH, El-Garhy HAS, Ramadan S (2016). Comparative genetic analysis among Moshtohor line rabbits and their parental lines using microsatellite markers.

[bib1.bib19] Emam AM, Afonso S, Azoz A, Mehaisen GMK, Gonzalez P, Ahmed NA, Ferrnand N (2016). Microsatellite polymorphism in some Egyptian and Spanish common rabbit breeds.

[bib1.bib20] Emam AM, Azoz A, Mehaisen GMK, Ferrnand N, Ahmed NA (2017). Diversity assessment among native middle Egypt rabbit populations in North upper- Egypt province by microsatellite polymorphism. World Rabbit Sci.

[bib1.bib21] Emam AM, Afonso S, Gonzalez-Redondo P, Mehaisen GMK, Azoz AAA, Ahmed NA, Fernand N (2020). Status and origin of Egyptian local rabbits in comparison with Spanish common rabbits using mitochondrial DNA sequence analysis. World Rabbit Sci.

[bib1.bib22] Evanno G, Regnaut S, Goudet J (2005). Detecting the number of clusters of individuals using the software structure: A simulation study. Mol Ecol.

[bib1.bib23] FAO (2011). Molecular genetic characterization of animal genetic resources.

[bib1.bib24] Scherf BD, Pilling D, FAO (2015). The Second Report on the State of the World's Animal Genetic Resources for Food and Agriculture.

[bib1.bib25] FAO (2019). Biodiversity and the livestock sector – Guidelines for quantitative assessment (Draft for public review).

[bib1.bib26] FAO (2021). FAO statistical data, Crops and livestock products.

[bib1.bib27] Fouzia BK, Homrani A, Ammam A (2017). Population structure and genetic diversity using microsatellite markers of four Algerian rabbit populations precludes hybridization with foreign breeds. South Asian J Exp Biol.

[bib1.bib28] Frankham R, Ballou J, Briscoe D, McInnes K (2002). Introduction to Conservation Genetics.

[bib1.bib29] Fuller SJ, Wilson JC, Mather PB (1997). Patterns of differentiation among wild rabbit populations Oryctolagus Cuniculus L. in arid and semiarid ecosystems of North-Eastern Australia. Mol Ecol.

[bib1.bib30] Gacem M, Lebas F (2000). Rabbit husbandry in Algeria, Technical structure and evaluation of performances.

[bib1.bib31] Hoban S, Archer F, Bertola L, Bragg J, Breed MF, Bruford MW, Coleman MA, Ekblom R, Funk WC, Grueber CE, Hand BK, Jaffé R, Jensen EL, Johnson J, Kershaw F, Liggins L, MacDonald A, Mergeay J, Miller J, Muller-Karger F, O'Brien D, Paz-Vinas I, Potter KM, Razgour O, Vernesi C, Hunter ME (2022). Global genetic diversity status and trends: Towards a suite of Essential Biodiversity Variables (EBVs) for genetic composition. Biol Rev.

[bib1.bib32] Holliday JA, Hallerman EM, Haak DC, Rajora OP (2018). Population Genomics.

[bib1.bib33] Hollingsworth PM, O'Brien D, Ennos RA, Ahrends A, Ballingall KT, Brooker RW, Burke T, Cavers S, Dawson IK, Elston DA, Kerr J, Marshall DF, Neaves L, Pakeman RJ, Trivedi C, Wall E, Wright F, Yahr R, Bean C, Blake D, Campbell R, Comont R, Finger A, Fraser K, Genney D, Hall J, Hannah A, Jehle R, Jones S, Kohn D, Llewellyn M, Lurz P, Macdonald I, McIntosh J, Mitchell R, O'Dell J, Page S, Pemberton J, Pérez-Espona S, Piertney S, Sime I, Thompson D, Ogden R (2020). Scotland's Biodiversity Progress to 2020 Aichi Targets: Aichi Target 13 – Genetic Diversity Maintained – Supplementary Report 2020.

[bib1.bib34] Iannella A, Peacock D, Cassey P, Schwensow N (2019). Genetic perspectives on the historical introduction of the European rabbit (Oryctolagus cuniculus) to Australia. Biol Invasions.

[bib1.bib35] Jochová M, Novák K, Kott T, Volek Z, Majzlík I, Tůmová E (2017). Genetic characterization of Czech local rabbit breeds using microsatellite analysis. Livest Sci.

[bib1.bib36] Kalinowski ST, Taper ML, Marshall TC (2007). Revising how the computer program CERVUS accommodates genotyping error increases success in paternity assignment. Mol Ecol.

[bib1.bib37] Karsli BA, Demir E, Fidan HG, Karsli T (2020). Assessment of genetic diversity and differentiation among four indigenous Turkish sheep breeds using microsatellites. Arch Anim Breed.

[bib1.bib38] Kettenring KM, Mercer KL, Adams CR, Hines J (2014). Application of genetic diversity-ecosystem function research to ecological restoration. J Appl Ecol.

[bib1.bib39] Lai FY, Ding ST, Tu PA, Chen RS, Lin DY, Lin EC, Wang PH (2018). Population structure and phylogenetic analysis of laboratory rabbits in Taiwan based on microsatellite markers. World Rabbit Sci.

[bib1.bib40] Ma Q, Wu B, Jiang J, Song Z (2020). Genetic Characterization of Selected Domestic Populations of Channel Catfish (Ictalurus punctatus) using Microsatellites. Pakistan J Zool.

[bib1.bib41] Mogharbi A, Mediouni MR, Ameur Ameur A, Azzi N, an dGaouar SBS (2021). Morphometric characterization of domestic rabbits (*Oryctolagus cuniculus domesticus *L.) in western Algeria. Genet Biodiv J.

[bib1.bib42] Mohamed EA, Abdelfattah MG (2018). Genetic Diversity Assessment Among Six Rabbit Breeds Using Rapd and Srap Markers. Egypt J Genet Cytol.

[bib1.bib43] Núñez-Torres OP, Almeida-Secaira RI (2022). Quantitative genetics: principles of farming in livestock production. J Selva Andina Anim Sci.

[bib1.bib44] Pavlova A, Beheregaray LB, Coleman R, Gilligan D, Harrisson KA, Ingram BA, Kearns J, Lamb AM, Lintermans M, Lyon J, Nguyen TTT, Sasaki M, Tonkin Z, Yen JDL, Sunnucks P (2017). Severe consequences of habitat fragmentation on genetic diversity of an endangered Australian freshwater fish: a call for assisted gene flow. Evol Appl.

[bib1.bib45] Peakall R, Smouse PE (2012). GenAlEx6.5: genetic analysis in Excel, Population genetic software for teaching and research – an update. Bioinformatics.

[bib1.bib46] Pritchard JK, Stephens M, Donnelly P (2000). Inference of population structure using multilocus genotype data. Genetics.

[bib1.bib47] R Development Core Team (2008). R: A Language and Environment for Statistical Computing.

[bib1.bib48] Sastry NSR (2023). The Indian Situation of Livestock Farming & Planetary Boundaries. Indian J Anim Prod Manage.

[bib1.bib49] Schmidt TL, Jasper M, Weeks AR, Hoffmann AA (2021). Unbiased population heterozygosity estimates from genome-wide sequence data. Methods Ecol Evol.

[bib1.bib50] Shevchenko Y, Kopylov K (2015). Genotyping of New Zealand White Rabbits by PCR-RFLP Markers. Agricultural Science and Practice.

[bib1.bib51] Sigsgaard EE, Jensen MR, Winkelmann IE, Møller PR, Hansen MM, Thomsen PF (2020). Population-level inferences from environmental DNA – Current status and future perspectives. Evol Appl.

[bib1.bib52] Teixeira JC, Huber CD (2021). The inflated significance of neutral genetic diversity in conservation genetics. P Natl Acad Sci.

[bib1.bib53] Van Loon EE, Cleary DFR, Fauvelot C (2007). ARES: software to compare allelic richness between uneven samples. Mol Ecol Notes.

[bib1.bib54] Woldvogel AM, Pfenninger M (2021). Temperature dependence of spontaneous mutation rates. Cold Spring Harbor Laboratory Press.

[bib1.bib55] Xia Q, Wang X, Pan Z, Zhang R, Wei C, Chu M, Di R (2021). Genetic diversity and phylogenetic relationship of nine sheep populations based on microsatellite markers. Arch Anim Breed.

[bib1.bib56] Zeroual A, Assani AA, Meddi H, Bouabdelli S, Zeroual S, Alkama R, Negm AM, Bouderbala A, Chenchouni H, Barceló D (2020). Water Resources in Algeria – Part I: Assessment of Surface and Groundwater Resources, Hdb Env Chem (97).

[bib1.bib57] Zerrouki N, Bolet G, Berchiche M, Lebas F (2005). Evaluation of breeding performance of a local Algerian rabbit population raised in the Tizi-Ouzou area (Kabylia). World Rabbit Sci.

