# Supplementary material for: Assessment of genetic diversity among native Algerian rabbit populations using microsatellite markers
Source: Arch Anim Breed. 2023 Jul 27;66(3):207–15. doi: 10.5194/aab-66-207-2023 (PMC10407306; doi:10.5194/aab-66-207-2023)
Supplement: The supplement related to this article is available online at: https://doi.org/10.5194/aab-66-207-2023-supplement. [file aab-66-207-supplement.pdf]

Supplement of Arch. Anim. Breed., 66, 207–215, 2023
https://doi.org/10.5194/aab-66-207-2023-supplement
© Author(s) 2023. CC BY 4.0 License.

Open Access
Archives Animal Breeding

Supplement of

Assessment of genetic diversity among native Algerian rabbit populations using microsatellite markers

Abdelbaki Bouhali et al.

Correspondence to: Abdelbaki Bouhali (bouhali.abdelbaki@gmail.com, a.bouhali@ens-lagh.dz)

The copyright of individual parts of the supplement might differ from the article licence.

Table S 1. Loci, and PCR reactions used in this study

Multiplex	Loci
**1**	INRACCDDV0108
	INRACCDDV0172
	INRACCDDV0176
	INRACCDDV0106
	INRACCDDV0139
	INRACCDDV0101
	INRACCDDV0203
	INRACCDDV0016

PCR reactions (Multiplex 1)

**Temperature**	95	95	63 - 55 (↓10)	72	95	55	72	60	10
**Time**	15 m	30 s	1 min	45 s	30 s	30 s	45 s	20 m	∞
**Cycles**			9x			38x			

Multiplex	Loci
**2**	INRACCDDV0089
	INRACCDDV0140
	INRACCDDV0087
	INRACCDDV0157
	INRACCDDV0119
	INRACCDDV0201

PCR reactions (Multiplex 2)

**Temperature**	95	95	61 - 55 (↓10)	72	95	55	72	60	10
**Time**	15 m	30 s	45 s	45 s	30 s	45 s	1 min	20 m	∞
**Cycles**			7x			37x			

Multiplex	Loci
**3**	SAT 3
	SAT 4
	SAT 5
	SAT 8
	SAT 7

PCR reactions (Multiplex 3)

**Temperature**	95	95	60	72	95	53	72	60	10
**Time**	15 m	30 s	30 s	45 s	30 s	30 s	45 s	20 m	∞
**Cycles**			38 x			7 x			

Multiplex	Loci
**4**	INRACCDDV0102
	SAT13
	INRACCDDV0169
	INRACCDDV0104
	INRACCDDV0205
	INRACCDDV0228
	INRACCDDV0192

PCR reactions (Multiplex 4)

Tcof1GH

**Temperature**	95	95	59 - 54 (↓10)	72	95	54	72	95	53	72	60	10
**Time**	15 m	30 s	30 s	30 s	30 s	45 s	45 s	30 s	30 s	45 s	20 m	∞
**Cycles**			6x			29x			8x			

Multiplex	Loci
**5**	INRACCDDV0313
	INRACCDDV0182
	INRACCDDV0040
	INRACCDDV0185
	INRACCDDV0259

PCR reactions (Multiplex 5)

**Temperature**	95	95	63 - 56 (↓10)	72	95	56	72	60	10
**Time**	15 m	30 s	45 s	45 s	30 s	30 s	45 s	20 m	∞
**Cycles**			8x			38x			

Group	Genes
**6**	KLH13
	PR0C
	EDNRA
	GPR64

**Temperature**	95	95	66 - 62 (↓10)	72	95	62	72	60	10
**Time**	15 m	30 s	45 s	45 s	30 s	30 s	45 s	20 m	∞
**Cycles**		5x			38x				

Group	Genes
**7**	CYTC
	ARH
	HPRT
	INRACCDDV0342

**Temperature**	95	95	67 - 60 (↓10)	72	95	60	72	95	53	72	60	10
**Time**	15 m	30 s	30 s	45 s	30 s	30 s	45 s	30 s	30 s	45 s	20 m	∞
**Cycles**		8x			30x			8x				

Group	Genes
8	BCDO2
	TYR
	GPC4
	MSTN
	IGF1
	FGF5
	SOX9
	GHRH
	GH
	IGF1R

**Temperature**	95	95	63 - 56 (↓10)	72	95	56	72	60	10
**Time**	15 m	30 s	45 s	45 s	30 s	45 s	45 s	20 m	∞
**Cycles**		9x			31x				

Group	Genes
**9**	BDNF
	HTR1A
	RSPO2
	TCOF1
	STMN1
	MSX2
	HINT1
	CLOCK
	HTR2A

**Temperature**	95	95	65 - 58 (↓10)	72	95	58	72	95	53	72	60	10
**Time**	15m	30s	45s	45s	30s	45s	45s	30s	45s	45s	20m	∞
**Cycles**		8x			30x			8x				

Group	Genes
**10**	BMP15
	LYNX2
	TSHR B*
	DRD2
	TPH2
	FEV

**Temperature**	95	95	69 - 61 (↓10)	72	95	61	72	60	10
**Time**	15 m	30 s	1 min 30 s	45 s	30 s	45 s	45 s	20 m	∞
**Cycles**		9x			38x				

11

SLC6A4, KRT71, SLC6A1, HTR1B

**Temperature**	95	95	69 - 61 (↓10)	72	95	61	72	60	10
**Time**	15 m	30 s	1 min 30 s	45 s	30 s	45 s	45 s	20 m	∞
**Cycles**		9x			38x				

12

KISS1, PAX8, SERP, DRD3, TSHB, ESR, FOXE1

**Temperature**	95	95	69 - 62 (↓10)	72	95	62	72	95	53	72	60	10
**Time**	15 m	30 s	45 s	45 s	30 s	45 s	45 s	30 s	45 s	45 s	20 m	∞
**Cycles**		8x			30x			8x				

13

TSHR A*, GHR, KITLG, MED12, ALB, CALCB, AVP

**Temperature**	95	95	69 - 62 (↓10)	72	95	62	72	95	53	72	60	10
**Time**	15 m	30 s	45 s	45 s	30 s	45 s	45 s	30 s	45 s	45 s	20 m	∞
**Cycles**		8x			30x			8x				

14

Amot, ext

**Temperature**	95	95	65 - 60 (↓10)	72	95	60	72	95	53	72	60	10
**Time**	15 m	30 s	30 s	45 s	30 s	30 s	45 s	30 s	30 s	45 s	20 m	∞
**Cycles**		6x			30x			8x				

15

cryl

**Temperature**	95	95	67 - 59 (↓10)	72	95	60	72	60	10
**Time**	15 m	30 s	45 s	45 s	30 s	30 s	45 s	20 m	∞
**Cycles**		9x			38x				

Minutes (m), seconds (s).

Table S 2. Genetic variability of 85 microsatellite loci in seven Algerian Rabbit populations

Locus	Na	Pa	H_o ±SE	H_e ±SE	*PIC*	HWE
INRACCDDV0101	5	1	0.367±0.025	0.418±0.036	0.797	*
INRACCDDV0106	5	1	0.636±0.037	0.700±0.005	0.695	*
INRACCDDV0108	4	0	0.468±0.038	0.514±0.030	0.459	**
INRACCDDV0139	5	0	0.538±0.052	0.611±0.029	0.591	*
INRACCDDV0016	5	0	0.561±0.037	0.666±0.019	0.662	*
INRACCDDV0172	8	3	0.551±0.026	0.608±0.034	0.593	*
INRACCDDV0176	9	3	0.147±0.030	0.183±0.038	0.183	NS
INRACCDDV0203	7	2	0.331±0.054	0.560±0.047	0.553	*
INRACCDDV0119	7	3	0.520±0.022	0.602±0.014	0.575	*
INRACCDDV0140	4	0	0.514±0.042	0.538±0.029	0.466	* *
INRACCDDV0157	7	2	0.336±0.043	0.586±0.046	0.610	*
INRACCDDV0201	7	1	0.418±0.037	0.495±0.038	0.454	* *
INRACCDDV0087	11	2	0.572±0.045	0.817±0.012	0.846	*
INRACCDDV0089	5	1	0.363±0.051	0.407±0.046	0.390	* * *
Sat3	8	1	0.591±0.041	0.611±0.032	0.611	*
Sat4	9	2	0.621±0.063	0.731±0.027	0.744	*
Sat5	8	1	0.551±0.041	0.584±0.025	0.597	*
Sat7	8	3	0.451±0.039	0.544±0.024	0.522	*
Sat8	5	1	0.602±0.023	0.630±0.018	0.602	*
INRACCDDV0102	9	3	0.718±0.045	0.760±0.014	0.754	*
INRACCDDV0104	5	0	0.536±0.037	0.571±0.031	0.557	*
INRACCDDV0169	6	1	0.455±0.044	0.506±0.038	0.481	* *
INRACCDDV0192	8	1	0.529±0.041	0.577±0.043	0.579	*
INRACCDDV0205	16	1	0.407±0.042	0.592±0.032	0.601	*
INRACCDDV0228	4	0	0.414±0.072	0.474±0.056	0.174	NS
Sat13	6	0	0.710±0.016	0.664±0.015	0.642	*
INRACCDDV0182	8	4	0.461±0.052	0.592±0.023	0.556	*
INRACCDDV0185	5	1	0.493±0.053	0.525±0.051	0.489	* *
INRACCDDV0259	9	1	0.611±0.034	0.674±0.032	0.663	*
INRACCDDV0313	9	2	0.662±0.040	0.712±0.037	0.727	*
INRACCDDV0040	9	3	0.626±0.051	0.712±0.014	0.202	NS
EDNRA	6	1	0.403±0.043	0.483±0.024	0.439	* *
GPR64	4	0	0.585±0.044	0.627±0.021	0.574	*
KLH13	6	0	0.228±0.039	0.656±0.034	0.674	*
Proc	8	1	0.452±0.037	0.551±0.034	0.546	*
ARH	3	1	0.013±0.009	0.037±0.024	0.038	NS
CYTC	7	0	0.449±0.022	0.656±0.024	0.642	*
HPRT	7	0	0,229±0.038	0.569±0.048	0.581	*
mOC342	11	2	0.633±0.033	0.748±0.022	0.755	*
AMOT	5	0	0.247±0.037	0.553±0.032	0.336	* * *
EXT	4	1	0.423±0.037	0.513±0.024	0.470	* *
CRYL	8	2	0.451±0.051	0.489±0.034	0.473	* *
BC02	8	2	0.617±0.046	0.686±0.032	0.690	*
FGF5	5	1	0.413±0.057	0.486±0.032	0.431	* *
GH	5	1	0.390±0.063	0.377±0.055	0.369	* *
GHRH	6	0	0.429±0.055	0.528±0.020	0.710	*
GPC4	4	1	0.020±0.09	0.159±0.033	0.160	NS
IGF1	5	0	0.469±0.046	0.567±0.025	0.511	*
IGF1R	3	0	0.045±0.019	0.043±0.018	0.445	* *
MSTN	6	0	0.424±0.037	0.501±0.032	0.480	* *
SOX9	3	1	0.159±0.051	0.160±0.041	0.156	NS
TYR	7	2	0.397±0.058	0.613±0.035	0.593	*
BDNF	6	1	0.323±0.031	0.339±0.030	0.328	* * *
CLOCK	12	3	0.698±0.025	0.759±0.015	0.258	* * *
HINT1	14	3	0.592±0.039	0.735±0.022	0.746	*

HTR1A	3	0	0.176±0.046	0.327±0.044	0.936	*
HTR2A	2	1	0.000±0.000	0.013±0.013	0.113	NS
MSX2	7	3	0.448±0.068	0.485±0.037	0.444	* *
RSPO2	1	0	0.000±0.000	0.000±0.000	0.260	* * *
STMN1	4	2	0.100±0.033	0.092±0.030	0.192	NS
TCOF1	10	2	0.665±0.034	0.773±0.028	0.788	*
BMP15	4	1	0.132±0.023	0.318±0.044	0.309	* * *
DRD2	7	3	0.502±0.051	0.511±0.040	0.466	* *
FEV	5	2	0.321±0.046	0.376±0.040	0.155	NS
LYNX	9	2	0.584±0.035	0.758±0.006	0.763	*
TPH2	5	1	0.421±0.046	0.561±0.017	0.522	*
TSRH_B	4	1	0.407±0.017	0.426±0.017	0.354	* * *
HTRB1B	4	0	0.507±0.052	0.593±0.025	0.544	*
KRT71	10	1	0.457±0.055	0.746±0.017	0.770	*
SLC6A1	15	3	0.499±0.037	0.589±0.079	0.622	*
SLC6A4	7	2	0.423±0.044	0.482±0.034	0.433	* *
DRD3	9	5	0.449±0.037	0.605±0.025	0.553	*
ESR1	6	0	0.560±0.044	0.687±0.039	0.686	*
FOXE1	4	1	0.239±0.051	0.541±0.024	0.465	* *
KISS1	6	1	0.361±0.047	0.379±0.044	0.363	* * *
PAX8	4	0	0.046±0.020	0.045±0.019	0.145	NS
SERP	5	1	0.275±0.052	0.630±0.034	0.612	*
TSHB	5	1	0.485±0.055	0.491±0.034	0.477	* *
ALB	10	0	0.722±0.051	0.773±0.020	0.774	*
AVP	7	2	0.449±0.052	0.458±0.048	0.418	* *
CALCB	8	2	0.458± 0.069	0.507±0.065	0.518	*
GHR_B	5	1	0.573±0.011	0.626±0.013	0.599	*
KITLG_A	6	0	0.507±0.036	0.677±0.019	0.663	*
MED12_A	6	1	0.176±0.023	0.541±0.040	0.521	*
TSHR_A	6	0	0.477±0.051	0.569±0.023	0.512	*

Number of observed alleles per locus (Na), Number of private alleles per locus (Pa), mean observed and expected heterozygosity (H_o and H_e), polymorphism information content per locus (*PIC*), significance of Hardy-Weinberg Equilibrium (HWE): **P*<0.05; ***P*<0.01,*** *P*<0.001, NS: non-significant.
